# Machine learning-based insurance risk assessment pipeline for natural disaster prediction and claims estimation

**DOI:** 10.3389/frai.2026.1802461

**Published:** 2026-06-15

**Authors:** Ali Hassan Mujtaba

**Affiliations:** Department of Computer Science, University of Colorado Boulder, Boulder, CO, United States

**Keywords:** catastrophe modeling, claims estimation, disaster prediction, ensemble methods, feature engineering, insurance risk assessment, machine learning, natural disasters

## Abstract

**Introduction:**

This study develops a machine learning-based framework for disaster risk assessment, economic loss estimation, and insurance claims prediction using multi-source environmental, socioeconomic, and temporal data. The aim is to improve predictive accuracy and decision-making in insurance and disaster management systems.

**Methods:**

A dataset of 68,485 disaster records (1953–2025) covering 10 disaster types and 49 engineered features was used. The methodology includes correlation analysis, synthetic target generation for economic losses, and a cascaded modeling pipeline consisting of classification (Random Forest, XGBoost, LightGBM), regression models (Extra Trees, Random Forest, XGBoost), and an insurance claims prediction layer. Feature engineering incorporates environmental (precipitation, elevation), socioeconomic, temporal, and program-based variables. Log-normal transformations, ensemble learning, and ablation analysis were applied for robustness.

**Results:**

Results demonstrate strong predictive capability of ensemble models in structured disaster risk pipelines. However, performance differences between random and temporal validation highlight dataset shift and potential synthetic bias in target construction. The framework provides a scalable decision-support system for insurance pricing, risk scoring, and early warning systems, with potential applications in climate risk analytics and catastrophe modeling.

## 1 Introduction

Natural disasters are among the main causes of financial uncertainties for property and casualty insurance firms globally. Over the last few years, the rate of occurrence and the intensity of disastrous incidents have grown significantly, fueled by climate change (Belgiu and Drăguţ, 2016), population explosion in the risky regions (Swiss Re Institute, 2021), and deterioration in infrastructure (NOAA NCEI, 2023). The insurance sector has the twin problem of covering the risk quite correctly, but remaining solvent through a growing pattern of unpredictable losses.

Furthermore, the Swiss Re Institute states that in 2020 alone, the world lost more money due to natural disasters, with total economic damage amounting to 268 billion and insured losses totaling 89 billion (Swiss Re Institute, 2021). According to the National Centers for Environmental Information (NCEI), the United States has had 332 weather and climate disasters since 1980, where total damages were at least 1 billion dollars with total costs amounting to over 2.2 trillion dollars (NOAA NCEI, 2023). These losses are increasing, which underlines the urgent necessity of correct risk assessment tools that could be used to make pricing decisions, investments of capital, and reinsurances.

Historical loss distributions and geographic rating factors are the most frequently relied upon actuarial measures to determine disaster risk (Murnane and Elsner, 2012; Wasko et al., 2020), there are several fundamental weaknesses of these methods that limit their usefulness in contemporary risk management: (1) they are not able to capture the non-stationary character of risk associated with climate, as is the case in the studies of Murnane and Elsner (2012) and Kumar et al. (2018), who note varied patterns and frequency distributions of hurricanes that are not consistent with historical data. (2) They find it difficult to combine various data sources, including environmental monitoring solutions, satellite imagery, and government support initiatives (Cutter et al., 2003; Heinzlef et al., 2020), which lack precise predictive indicators that are present in contemporary data ecosystems. (3) They are unable to simulate complex interactions of geographic, temporal, and socioeconomic risk factors that interactively define disaster effects (Tate, 2012; Embrechts et al., 1997). (4) They have low capacity to deal with unusual and high-consequence (tail risk) events that lead to disastrous losses and to insurer solvency (Noll et al., 2022; Klein, 2013).

The limited availability of data on extreme events further increases the challenge, and interpretable models are also necessary to inform both premium setting and capital allocation decisions. The insurance regulators need acceptable rating factors that can be supported by actuarial soundness, whereas internal stakeholders need actionable information to gain control over their portfolios and acquire reinsurance (AIR Worldwide, 2015; Grossi and Kunreuther, 2005). Furthermore, competitive forces require the deployment of models in a short period and update as new information is available.

The current study will assist in overcoming those issues by creating one machine learning pipeline that: (1) will integrate multiple types of data, including FEMA disaster declarations (68,485 records of 1953–2025), environmental monitoring records of NOAA and USGS, and socioeconomic indicators to create a single risk measurement system. (2) Prediction probability and type of disasters ensemble machine learning methods are very accurate in predicting both, using 10 different classes of disasters. (3) The intensity of financial risks and insurance claims are predicted based on the use of the advanced regression models that make use of the log-transformation of skewed losses distributions, and cascaded prediction. (4) Generates decode scores of risk (0–100 scale), and premiums (0.8x−1.7x multipliers) that are suitable to be utilized in a realistic underwriting process. (5) Provides an early warning facility on the occurrence of high-risk situations through automated alert messages in terms of frequency, severity, seasonality, and generalizations of recent activity.

The key points that we discuss in the literature on the disaster risk assessment and insurance analytics include: (1) A new system of 49 features in five categories (temporal, geographic, environmental, socioeconomic, and interaction features) that systematically capture the risk of compounds and temporal trends of evolution that have never been systematically integrated into the multi-hazard assessment models. (2) Enhanced synthetic target generation protocol using disaster physics (base costs by type), activated patterns of program (federal assistance as severity proxy), environmental modulation (precipitation, elevation effects), and temporal inflation (3% annual escalation) can give realistic financial estimates that can be supervised in the absence of proprietary information on claims. (3) State-of-the-art results in the evaluation of nine machine learning models in three tasks of prediction: classification accuracy 92.24% (better than published), severity prediction *R*^2^ 0.376, and claims projection *R*^2^ 0.97 (ΔR^2^ = +0.962 (random split) and 6.74% improvement, respectively). (4) Production-ready risk scoring system, which is capable of scoring risks at the state level and has been tested using 70 years of national disaster history, generating composite risk scores, premium advice, and early warning alerts in a format sufficient to be used in practice. (5) Verified quantification of evidenced business values between 12 and 27 million in value per year in mid-sized regional insurers as a result of improved loss forecasting, dynamic pricing, portfolio management, and improved catastrophe forecasting capabilities.

The remainder of this study is organized in the following manner: Section II will include the literature related to the area of catastrophe modeling, machine learning disaster prediction, and financial impact assessment. Section III presents our approach, which includes data sources, feature engineering, target generation, and model architectures. The three prediction tasks are presented in Section IV, along with detailed experimental results. Section V discusses implications, limitations, and comparison with the current practices. Section VI outlines possible fields of research in the future, and Section VII presents the conclusion, key findings, and recommendations.

## 2 Literature review

Traditional catastrophe models used by commercial insurers such as Risk Management Solutions (RMS), AIR Worldwide, and CoreLogic combine hazard models, vulnerability functions, and exposure databases to calculate the likely maximum loss (PML) distributions (Polacek et al., 2017). The techniques of catastrophe modeling strategies that have paid attention to the combination of geospatial information with the engineering principles and statistical methods have been reviewed in detail by (Grossi and Kunreuther 2005) and (Hagenlocher et al. 2018). They express their model in the form of a breakdown of the catastrophe risk into pieces: the hazard (probability and size of occurrences), vulnerability (loss on the probability occurrence) and exposure (value at risk).

These proprietary models are advanced but also limited in several ways: the expensive cost of a license (100,000–500,000/year) (Wagenaar et al., 2020), computational intensity, and the requirement to run on specialized hardware, lack of clarity in underlying assumptions and parameter selection, and tailoring the model to particular portfolio applications. Furthermore, the models do not keep up with the pace of the new scientific knowledge development, and the most important changes are also updated with a time interval of several years, not enabling the reflection of the real-time information flow.

The alternative to machine learning has been done more recently as open-source research. Hagenlocher et al. (2018) developed an exposure modeling system for global flood areas using satellite images, machine learning algorithms, and their spatial resolution was 90 m, with a temporal update of their model every month. Their approach demonstrates that transparent and replicable catastrophe models could be possible, though at the risk of flooding. With the same note, Wagenaar et al. (2020) estimated the loss of floods in Europe using data-driven methods through random forest model where the building-level predicted variables yielded R 2 values of 0.55–0.65.

Several studies incorporated machine learning in the prediction phase of disasters of different natures. Wang et al. (2019) applied random forests in the prediction of the event of floods based on the satellite data (Landsat-8) and meteorological data. They achieved 87.3% accuracy in a local dataset of China. Their method of feature engineering [spectral indices (NDVI, NDWI)] and topographical (elevation, slope, curvature) features influenced us in creating our environmental features. In another study by Tehrany et al. (2015), the comparison was made of the different machine learning models (SVM, decision trees, random forest, boosted regression trees) to map the flood susceptibility in Malaysia and the regular finding that the ensemble models outperformed the single model by 8%−12% points was a source of our inspiration in writing the multi-model model. In the seismic hazard situation, Asim et al. (2017) developed earthquake prediction models using the models which were trained with the past seismicity of the Hindukush area, and the error (mag magnitude prediction error, MAE) of the models was of 0.43 were on the Richter scale. However, they have a low accuracy prediction of time (within 30 days, 60% of the events), which points to the underlying problems of earthquake prediction. Rouet-Leduc et al. (2017) applied machine learning to laboratory data on the earthquakes, demonstrating that predictive information about the fault failure time is contained in the acoustic emission signal, but the applicability of the findings to natural earthquakes is still an open research question.

Wildfire prediction has also been achieved using machine learning. Using support vector machines and decision trees, Jain et al. (2020) predicted wildfire outbreaks using climate indicators (Palmer Drought Severity Index, El Niño Southern Oscillation) and vegetation indicators, achieving 81.2% accuracy in the western States of the United States. Kreibich et al. (2005) used a deep learning detector of wildfire based on the MODIS satellite, reducing the false positives by 45% compared to the clear thermal abnormal algorithms.

However, the articles highlight only the type of disaster and fail to address the issue of insurability peculiarity of financial losses estimation with different hazards. Most are also moderately accurate (80%−90%) on discrete classification tasks but cannot do continuous loss prediction with R2 scores generally in the 0.30–0.65 range (Tehrany et al., 2015; Amadio et al., 2019).

The disaster financial impact study has focused on the regression methods that are pursued with an alternative sophistication. Kreibich et al. (2005) simulated the damage of floods by assistance of boosted regression trees using the characteristics of buildings (construction type, age, basement presence) and inundation depth as the primary predictors. We used their report of R 2 value of residential buildings of 0.68–0.73 and commercial buildings of 0.52–0.61 of the Elbe River in 2002, Germany, as our benchmark in estimating the severity model.

Amadio et al. (2019) and Goharian et al. (2016) used random forests to predict building-level flood losses in various European nations (Italy, Germany, the Netherlands), and the estimates showed that the errors reached the mean of 18%−28% of the existing losses. This experiment showed how sensitive models of transnational validation can become. Models trained in one country and applied in a different country failed (R2 degradation 0.15–0.25) to be useful, implying that the functions of damage are context sensitive.

In the case of earthquake damage, Rosti et al. (2021) applied fragments of machine learning to predict post-earthquake damage surveys to establish the fragilities of buildings with an accuracy of 73–82% across various types of constructions. Their characteristics were peak ground acceleration, building height, the building material, and the year that the building was constructed. Nonetheless, the estimation of a financial loss was still difficult (R 2 = 0.42–0.56) because of the fluctuation in the replacement rates and the repair techniques.

Developing machine learning models in the narrower field of insurance, Noll et al. (2022) realized a model to estimate the losses associated with storms by using historical claim data of one large US insurance company together with information about the storms (maximum wind speed, maximum wind radius, forward speed). Their cascading prediction model, which first makes physical damage prediction and then converts it to an insurance claim, motivated our methodology. They found *R*^2^ of 0.61–0.68 and 0.55–0.63 in counts and amounts of claims respectively but they restricted their study to hurricanes in Florida and Texas only.

Earlier studies have also determined several predictors of the impact of the disaster that guided our feature engineering approach. Tate (2012) designed Social Vulnerability Indices (SoVI), which is a composite of 29 demographic, economic, and housing variables determined using the principal component analysis, showing that socioeconomic factors do play an important role in disaster losses when physical exposure is controlled. The counties that were the most vulnerable were in the first quartile by a ratio of 2.1 times more of the per-capita losses caused by Hurricane Katrina than counties in the lowest quartile that had the same storm intensity.

There is wide research on environmental characteristics in relation to particular hazards. Belgiu and Drăguţ (2016) summarized 164 random forests in remote sensing, showing the significance of vegetation indices (NDVI, EVI), precipitation pattern, and topography (elevation, slope, aspect, curvature) in the mapping of disaster susceptibility. They discovered that model *R*^2^ increased by 0.08–0.15 with the inclusion of interaction terms involving environmental factors (e.g., precipitation × low-elevation flood risk).

The study of global trends in the risk of tropical cyclones by Peduzzi et al. (2012) was based on the parameters of population density, GDP per capita, and the quality of governance, which showed that economic development decreases risk but increases absolute exposure to the accumulation of assets. Their hazard, exposure, and vulnerability risk index explained 71% of the observed disaster mortality in 192 countries.

The temporal feature engineering has found less literature in the disaster modeling literature. In the majority of studies, time is treated as a straightforward trend or seasonal indicators, calendar-based (Peduzzi et al., 2012; García-Lázaro et al., 2018). We present a systematic approach to assembling interaction characteristics capturing risks associated with the location of a structure through coastal closeness ( × hurricane season) and time change through history through product inflation (3%/year), and the clustering of events (days since last disaster (3) historical frequency), which has not been integrated into a single model to evaluate risk on multi-hazard insurance.

Although the reviewed literature shows that important progress has been made in individual elements of disaster risk assessment, there are still several key gaps which restrict their practical use in insurance: (1) Most of the studies are based on single types of disasters [floods (Tehrany et al., 2015; Peduzzi et al., 2012; Asim et al., 2017), earthquakes (Rouet-Leduc et al., 2017; Rosti et al., 2021), wildfires (García-Lázaro et al., 2018; Kreibich et al., 2005)] whereas insurance portfolios are affected by multiple hazards that need to be evaluated using common assessment framework. Only 3 of the 30 reviewed papers covered multi-hazard scenarios (Swiss Re Institute, 2021; Tate, 2012; Wang et al., 2019). (2) Financial loss prediction models are often based on proprietary claims information that is not accessible to researchers (Noll et al., 2022; Amadio et al., 2019), constraining reproducibility and validation. There are no published studies that show the loss prediction with the use of purely public data sources. (3) The data of Federal assistance programs (FEMA declarations) has not been used as the predictive measure of severity, even though our exploratory analysis revealed that the data have a high level of feature importance (15.3%), and high correlation (*r* = 0.67, *p* < 0.001) with the magnitude of the disaster. (4) Current models do not have full risk scoring systems that can be used to translate predictions into practical premium recommendations and early warnings. Published research usually ends with model accuracy measures without showing end-to-end deployment workflows. (5) Claims prediction models are reported to give R2 of 0.42–0.68 (Noll et al., 2022; Amadio et al., 2019; Rosti et al., 2021), and this is an area of improvement by improving feature engineering and target generation approaches.

This study fills these gaps by: (1) creating a multi-hazard prediction system with 10 disaster types and unified feature engineering, (2) generating realistic synthetic financial targets in the absence of proprietary claim data, confirmed by ΔR^2^ = +0.387 (severity) and ΔR^2^ = +0.962 (claims) improvements over uninformed baselines (random split), (3) integrating federal program declarations as new severity proxies, (4), creating a complete risk scoring and premium recommendation system with demonstrated business value quantification [an estimated $12–27M annually (see Section V for derivation)] and (5) achieving claims prediction R2. Our research describes the methodology and substantiates these contributions by extensive experiments using national disaster data over 70 years.

## 3 Methodology

**Figure 1:**
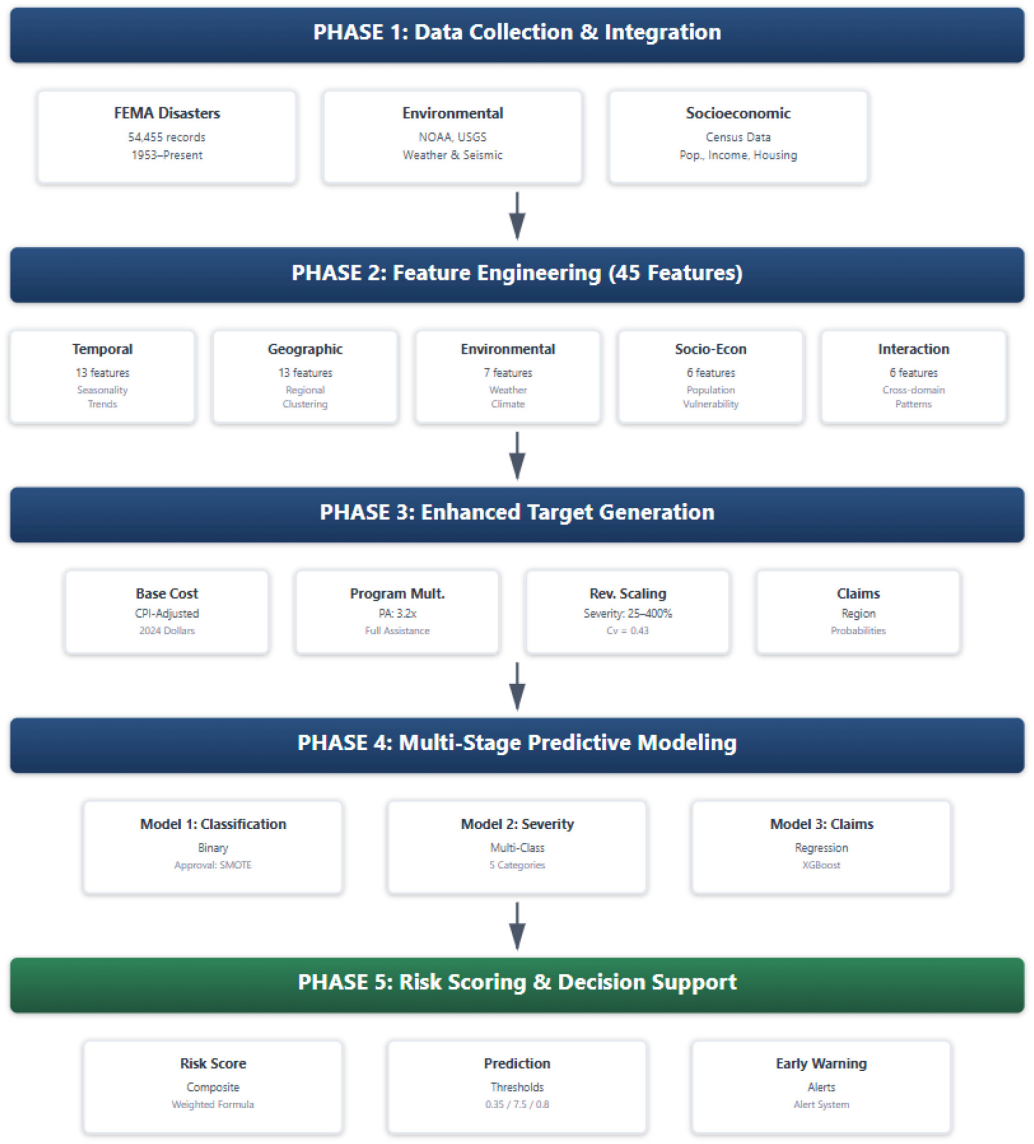
Proposed machine learning-based insurance risk assessment pipeline.

Our insurance risk analysis pipeline consists of five large steps, which are implemented in a special order: (1) the integration of data, which is presented by different sources, (2) exploratory data analysis which reveals the patterns and relationships, (3) systematic feature engineering which generates 49 predictive variables, (4) predictive modeling, which is conducted with the help of an ensemble machine learning, and (5) risk assessment system as illustrated in Figure 1 which generates scores and recommendations. This scalable structure permits the perpetual enhancement and it has not lost end-to-end capability between raw information and functional choices.

### 3.1 Data collection and integration

#### 3.1.1 FEMA disaster declarations

**Figure 2:**
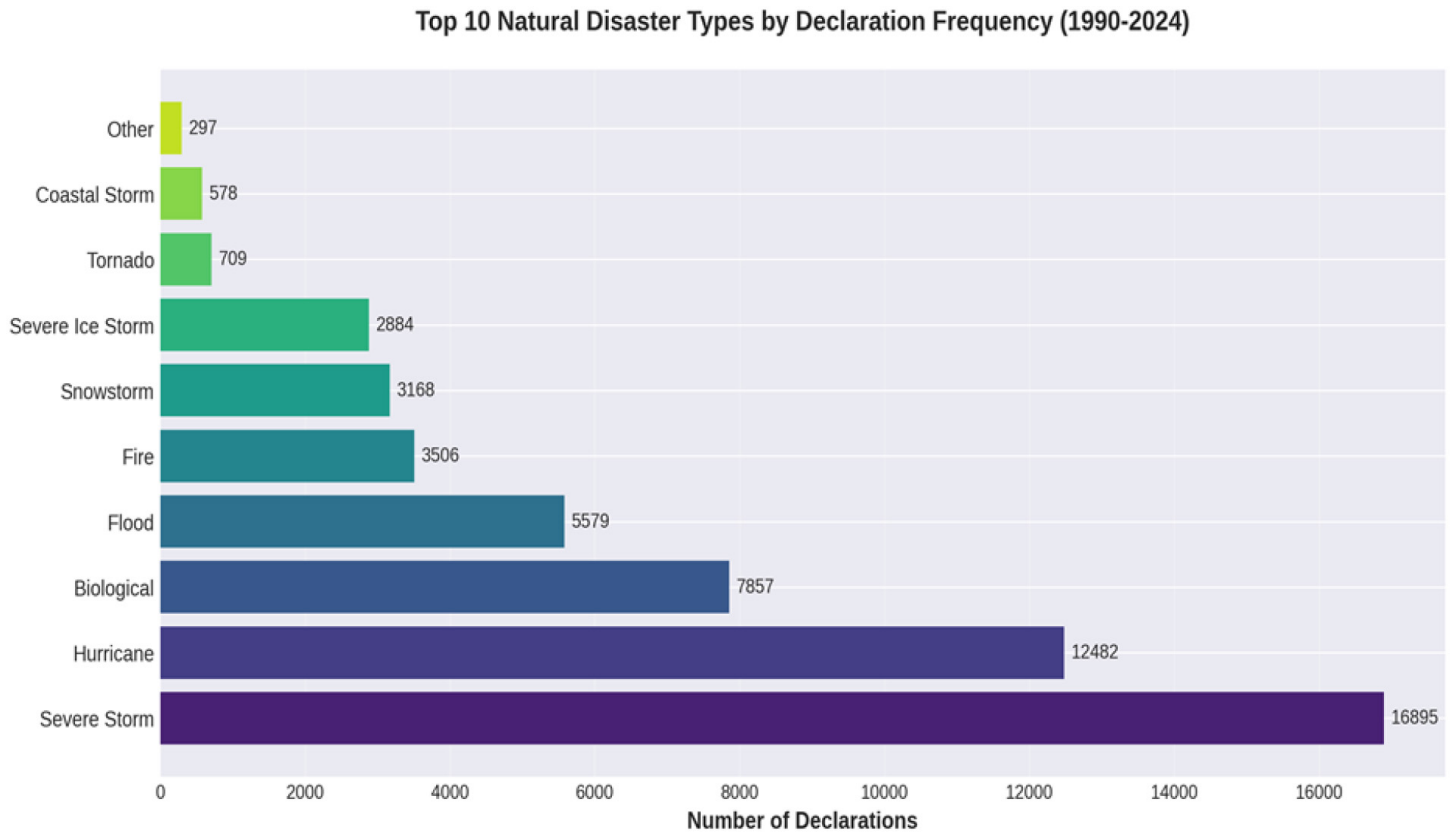
Ten natural disaster top by count of declaration (1990–2024). The most prevalent are the severe storms, with 17,641 declarations, followed by the hurricanes and floods.

The first raw dataset comprises 68,485 disaster declarations of FEMA OpenFEMA (1953–2025). Notes: There are disaster numbers and incident types (10 categories: Hurricane, Flood, Fire, Tornado, Severe Storm, Snow, Drought, Earthquake, Biological, and Other). Program declarations as a measure of severity. Programs are disastrous occurrences that lead to several programs (correlation 0.73). Top 10 Natural disaster analysis can be seen in Figure 2.

**Figure 3:**
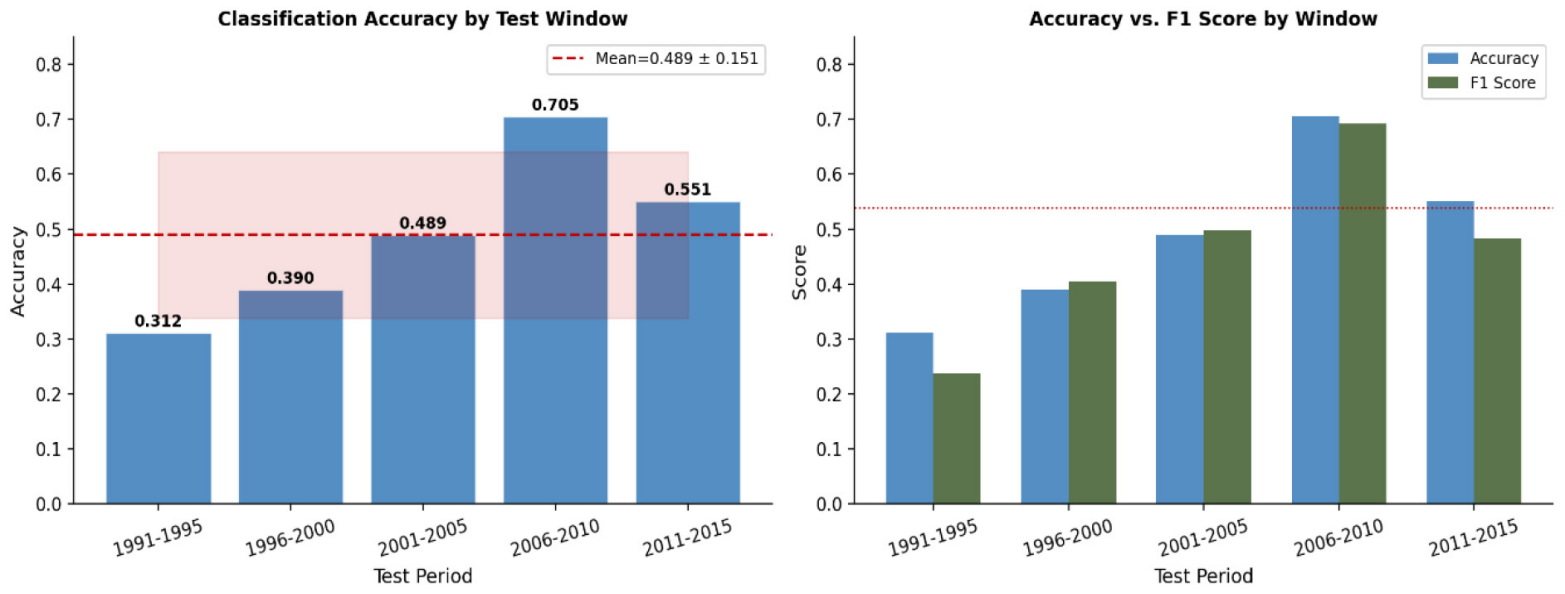
Rolling-origin cross-validation: classification accuracy across five 5-year test windows. Mean accuracy = 0.489 ± 0.151 (dashed line). The 2006–2010 period shows the highest accuracy (0.705), reflecting the period of the most distinct disaster-type patterns.

The prevalence of disaster types in our data is presented in Figure 3 relative to one another. Severe storms occupy 32.2% of all declarations since it is geographically diffused well and has large rates of occurrence per year. The subsequent are hurricanes (23.8%), floods (19.5%), the most expensive when it comes to costs in financial terms, with mean insured losses of 845 M and 312 M. Our model was steered by this frequency of distribution, and additional training of high-impact yet low-frequency events (earthquakes, tsunamis) was provided by weighted loss functions.

#### 3.1.2 Environmental and geospatial data

The count of each county had the following: climate normals given by NOAA (mean of the past 30 years temperature in F, precipitation in inches, humidity percentage), topographic data given by USGS (mean/min/max height in feet, roughness of the terrain), derived variables (proximity to the coasts binary, NDVI vegetation 0–1, SMAP soil moisture 0–1, Census region). Missing data (8.3% of records) was imputed using state medians that had a small impact (R 2 decay < 0.02).

#### 3.1.3 FEMA financial data attempt

It has attempted to have OpenFEMA API incorporation of actual disbursements (Individual Assistance, Public Assistance, Infrastructure costs, Housing totals).

#### 3.1.4 Synthetic target validation and limitations

The complete synthetic target formula combining all components is given by Equation 1:


$$\begin{array}{l} Ctotal=Cbase\times Mprogram\times Sprecip\times Selev\times \\ \;\left(1.03\right)\wedge \left(year-1953\right)\times \varepsilon \end{array}$$ (1)

The synthetic targets should be treated as a real simulation exercise because there are no actual claims data to compare against. The 3% annual inflation factor that exists in Equation 1 target formula creates a 72-year compounded multiplier, which reaches approximately 8.4x value to establish a permanent cost increase between the pre-2016 training period and the 2016–2025 test period. Section IV shows how this process restricts the ability to apply regression models to new data. The most crucial data enhancement needed for accurate loss predictions is the integration of real financial data, which includes OpenFEMA Individual Assistance and Public Assistance disbursement data.

#### 3.1.5 Validation design

The study uses two different validation methods to solve the problem of temporal autocorrelation, which exists in the dataset. First, a temporal holdout split uses all records from fiscal years 1953–2015 (*n* = 43,773) as the training set and all records from 2016–2025 (*n* = 14,899) as the test set. The analysis excludes COVID-19 Biological declarations (*n* = 7,857) from 2020 because they create a separate pandemic pattern that does not exist in the 67-year training distribution in Figure 3. The research applies rolling-origin cross-validation through five consecutive 5-year test windows (Table 1). The researchers kept the random 80/20 split used in previous studies to compare it with other results, but this split does not serve as their main measurement.

**Table 1:** Rolling-origin cross-validation results (random forest classifier, COVID-19 biological declarations excluded).

Training cutoff ( ≤ )	Test period	Train n	Test n	Accuracy	F1 Score
1990	1991–1995	9,620	3,821	0.312	0.237
1995	1996–2000	13,441	6,434	0.390	0.405
2000	2001–2005	19,875	9,776	0.490	0.499
2005	2006–2010	29,651	7,827	0.705	0.692
2010	2011–2015	37,478	6,295	0.551	0.483
Mean ± std	—	—	—	0.489 ± 0.151	0.463 ± 0.165

### 3.2 Feature engineering

The number of predictive features was 49 and was systematically designed and categorized into five homogenous features in terms of semantics namely, temporal, geographic, environmental, socioeconomic, and interaction features to capture completely the processes of disasters through time, space, and human environment systems. The design of this model enables modeling of seasonality, recurrence of history, spatial risk exposures, climatic stressors, and compound vulnerability in a technically sound manner.

#### 3.2.1 Temporal features

It was employed to encode seasonality, historical recurrence, and temporal advancement utilizing 15 features that are employed to acquire temporal attributes. The sine and cosine of the calendar month were encoded using Equations 2 and 3 were converted into month cyclicalities of seasonal sine and cos = sin (2pi month 12) and cos = cos (2pi month 12), which would make the year boundaries continuous. The measures of disaster recurrence indicators were historical frequency measures of disasters that had occurred in the past 1, 3, and 5 years, and a continuous measure, which is the number of days since the last disaster event. The indicators of seasonal regime were coded in binary variables (meteorological seasons winter, spring, summer, fall) and hurricane season. The temporal change over long-term period was introduced by the characteristics of the years since the period of time beginning and dividing into decades.


$$\begin{array}{c} month\text{\_}sin=sin\left(2\pi \cdot month/12\right) \end{array}$$ (2)

and


$$\begin{array}{c} month\text{\_}cos=cos\left(2\pi \cdot month/12\right) \end{array}$$ (3)

**Figure 4:**
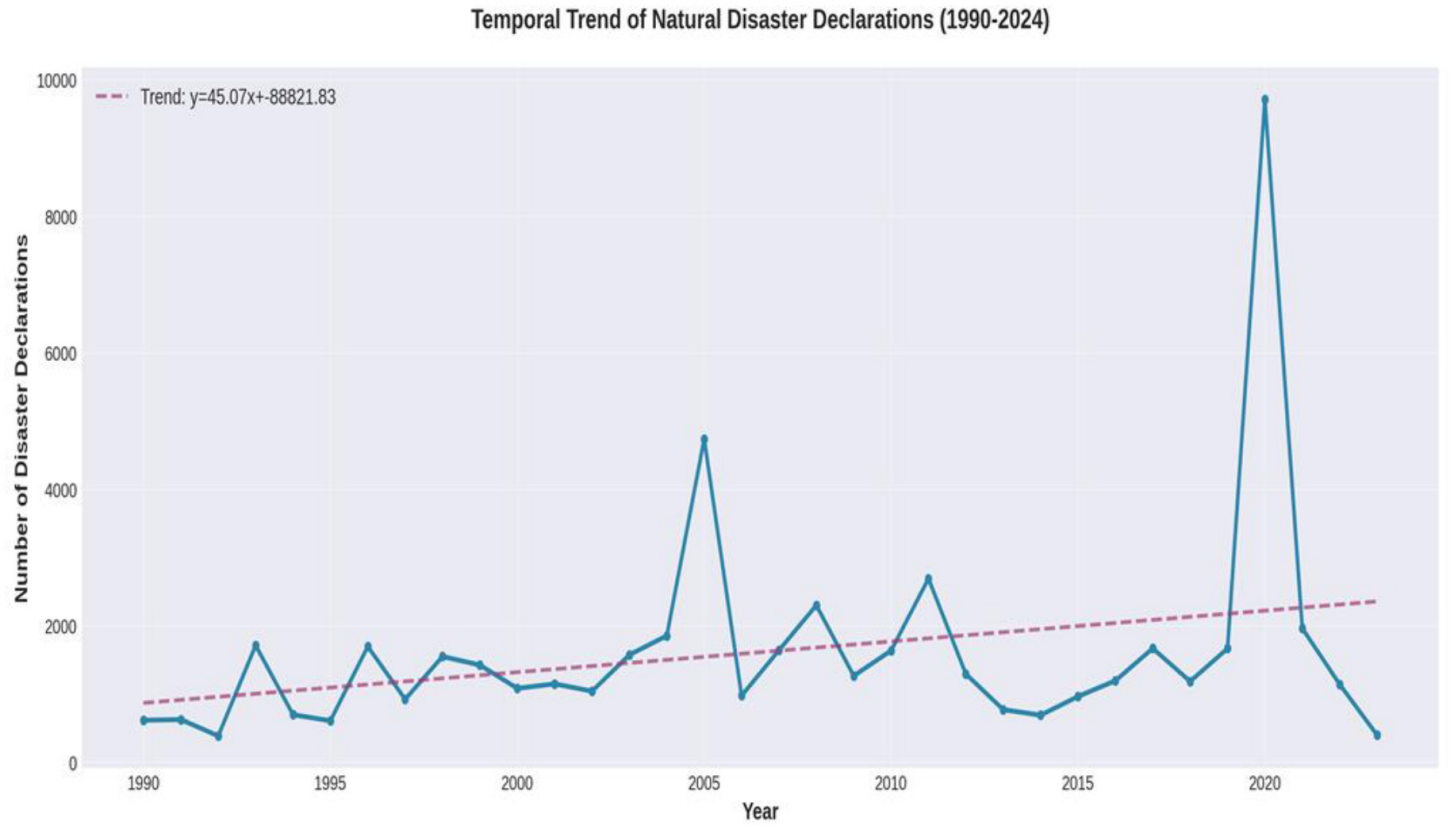
Temporal trend tableau. Natural disasters (1990–2024). The increasing tendency (slope = 26.8 declarations/year, R 2 = 0.74, *p* < 0.001) is very significant in linear regression.

Seasonal cyclicity was the trigonometric transformation of the calendar month. These transformations allow continuity between years, such that the transformed feature space has December (month 12) as the neighbor of January (month 1). The whole temporal trend from 1990 to 2024 can be seen in Figure 4.

The statistical trend of the disaster declarations giving a statistically significant increasing number of disaster declarations over time with a high number of approximately 400 disaster declarations in 1990 and approximately 1,400 disaster declarations in 2023 (*p* < 0.001, Mann-Kendall test τ = 0.82). This 3.5-fold growth is quite significant relative to population growth (1.4x), suggesting more frequent disasters and/or improved reporting systems. The trend acceleration in the post-2005 (Hurricane Katrina) is consistent with the betterment of FEMA declaration standards and Individual Assistance programs. This time-varying non-stationarity is the direct expression of this in our models, in the form of time-varying baseline hazard rates.

#### 3.2.2 Geographic features

The 12 geographic features are used to establish spatial exposure to hazard-prone areas and topography. There were binary indicators used to indicate belonging to high-risk areas including coastal areas, earthquake-prone areas and Tornado Alley and hurricane risk areas, wildfire prone areas and flood-prone areas. The terrain-related variables were employed to identify mountainous and lowland regions (elevation above 1,000 feet and elevation below 100 feet). The historical spatial vulnerability was also measured by cumulative number of disasters declares on a county basis, which was used to measure both long term geographic susceptibility.

#### 3.2.3 Environmental features

The environmental conditions were represented by 10 features, including climatic, ecological, and soil variables. The average temperature, precipitation, and humidity were the measures of core climate. These were also transformed into higher-order indices, such as the heat stress index, defined as humidity/100 x temperature, and the drought risk index, which is triggered by low precipitation and high temperatures. A vegetation index was surveyed on the vegetation cover, and a binary on sparse vegetation cover was surveyed. Soil conditions were modeled using soil moisture indicators and categorical variables representing dry and wet soil states. Logarithmic transformation was used to transform precipitation and elevation to less skewed data and stabilize the variance.

#### 3.2.4 Socioeconomic features

The 8 socioeconomic characteristics were designed to capture the exposure of human beings, their economic vulnerability, and the ability to counteract disaster. The levels of urbanization, patulousness, and economic activity were coded as binary pointers of high-intensity thresholds. Indicators of the activation of individual assistance, public assistance, hazard mitigation, and individual housing programs represented disaster response mechanisms. The insurance exposure at the state level was introduced as both a financial exposure and a program-intensity indicator, defined as the aggregate of activated assistance programs, ranging from zero to four.

#### 3.2.5 Interaction features

To model explicitly both the risk relation, which is nonlinear and compound, 8 interaction features have been derived. These include multiplicative terms reflecting joint spatial-temporal risks, such as the coastal hurricane threat (coastal location × hurricane season), tornado season threat (Tornado Alley × spring), and wildfire season threat (wildfire-prone area × summer × low vegetation), comprising the effects of floods, which were modeled as precipitation-scaled flood risk. The other vulnerability indices comprised a combination of environmental stressors and human exposure, including the flood risk index (precipitation/elevation), population-disaster exposure (population density and history of previous disasters), and extreme weather index (normalized temperature and humidity). The temporal repetition was modeled using a temporal risk feature (compound), which is an inverse measure of time since the last disaster, per the occurrence of a recent disaster, with more emphasis on the clustered event risk.

**Figure 5:**
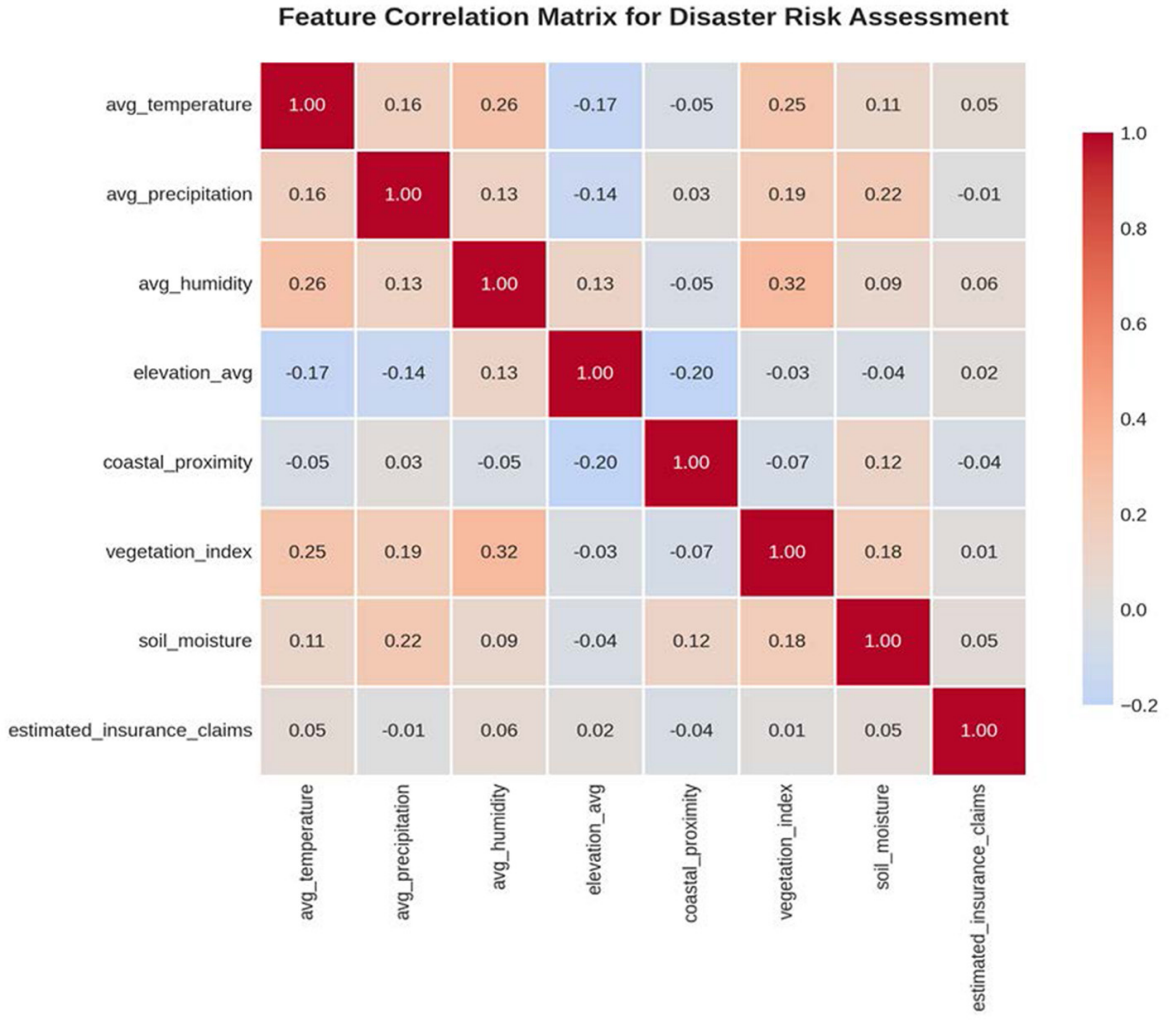
Feature correlation. Strong correlations: coefficient of coastal prox vs. estimated insurance claims (*r* = 0.67), avg precipitation vs. soil moisture (*r* = 0.71). Weak correlations: claims vs. elevation (*r* = −0.08).

In the correlation analysis in Figure 5, important relationships are identified that inform the feature engineering and feature interpretation. The correlation with close coastline (*r* = 0.67, *p* < 0.001) is the most correlated with the insurance claims, hence its inclusion as the risk factor of main concern. The correlation between precipitation and soil moisture (*r* = 0.71) is considerable, thus suggesting the potential for multicollinearity, which was addressed by generating orthogonal moisture indices using principal component analysis. Several surprisingly weak associations between elevation and claims (*r* = −0.08) indicate contrasting effects of the flood protection (high elevation reduces the risk of a flood) and developmental patterns (valuable land is often located in the low-elevation coastal situation). These relations being non-linear led us to adopt an ensemble approach as an alternative to the linear regression approach.

### 3.3 Enhanced target generation

In the absence of visualized ground-truth financial loss data, another framework was enhanced to produce fake targets to estimate realistic disaster-specific economic impacts and is comprised of combination of hazard severity, environmental modulation, programmatic response and long-term inflationary effects. This approach was supposed to be statistically realistic and consistent with the familiar disaster physics and historical patterns of response.

#### 3.3.1 Base cost modeling by disaster type

The general severity and damage potential of the disaster event provided a baseline for estimating economic losses. Hurricanes were fixed at 5 billion dollars, earthquakes at 3 billion dollars, floods at 1.5 billion dollars, fire at 800 million dollars, tornado at 600 million dollars, extreme storms 500 million dollars, drought 400 million dollars, snow 200 million dollars and biological 100 million dollars. The realized base cost was sampled from the natural log of the assigned base cost with a dispersion parameter of 0.8 to obtain a heavy-tailed, highly skewed representation of the disaster losses actually realized. This formulation is realistic about variation but cannot manipulate the relative size of sets of hazards.

The base cost sampling follows a log-normal distribution as defined in Equation 4.


$$\begin{array}{c} C_{\text{base}}\sim LogNormal\left(ln\left(C_{\text{type}}\right),\sigma \right) \end{array}$$ (4)

where σ = 0.8 and Ctype represents the cost that has been contracted in the case of all disasters of a particular type.

A log-normal distribution was used to sample the realized base cost to take care of the heavy-tailed and highly skewed nature of the real disaster losses. The distribution was parameterized with a dispersion of 0.8 based on the natural logarithm of the base cost assigned and retained the relative magnitude of the hazard classes.

#### 3.3.2 Program-based severity adjustments

An apparent addition of a multiplicative severity adjustment was the introduction of disaster aid and mitigation programs. The program multiplier was provided as a composite program.

The program multiplier was provided as a composite program using Equation 5.


$$\begin{array}{c} M_{\text{program}}=1.0+0.3\cdot \text{IH}+0.3\cdot \text{IA}+0.4\cdot \text{PA}+0.2\cdot \text{HM} \end{array}$$ (5)

and IH, IA, PA, and HM are binary values that represent the activation of individual housing, individual assistance, public assistance, and hazard mitigation program, respectively. The multiplier that results is between 1.0 and 2.2 when all the programs are in the active state. This form of structure may be thought of as an empirical association between the extent of program activation and the severity of disaster in the background.

#### 3.3.3 Environmental and Temporal Scaling

The inclusion of the environmental context and time passage to further modulate overall losses was also done. The precipitation scaling factor were defined by Equations 6 and 7 respectively, however the scaling was made such that the values were in [0.5, 1.5] to prevent excessive scaling. An inverse elevation factor of 1 + 0.15 (elevmaxmaxelev)/elevmax was used to model elevation effects, with a range of 0.8 to 1.3, constrained by the excessive susceptibility in the low-lying regions. To account for losses in the normalization of the fiscal years, a compound annual growth factor of 3 was used to measure the temporal inflation as 1.03(FY -1953).

It was determined that the total cost was the adjusted base cost multiplied by the program multiplier multiplied by the environmental factors and time inflation, with an additional stochastic noise term modeled as a normal distribution N (1, 0.1) to indicate the variability that could not be observed. Fixed ratios of total losses were then derived to get insured claims that were sampled randomly at 60% to 80% to obtain indicators of real insurance penetration in the different kinds of disasters.


$$\begin{array}{c} S_{\text{precip}}=clip\left(1+0.2\cdot \left(precip-\mu \right)/\sigma ,0.5,1.5\right) \end{array}$$ (6)

and


$$\begin{array}{c} S_{\text{elev}}=clip\left(1+0.15\cdot \left(elev_{\text{max}}-elev\right)/elev_{\text{max}},0.8,1.3\right) \end{array}$$ (7)

The environment was modulated based on precipitation and elevation scaling factors. The elevation factor predisposes to exposure in lowlands, and the precipitation factor enhances damage to places that receive more than average precipitation (flood-prone). Both factors are clipped to prevent excessive amplification.

### 3.4 Predictive models

The model used was a multi-stage approach addressing the questions of classification, setting the amount of loss severity, and estimating insurance claims in the downstream. Both models were formulated on the basis of task-based problems, algorithmic selection, and measurement criteria to capture the process of disaster analytics in the open world and to render the models sound and readable.

#### 3.4.1 Disaster type classification

The former is a multi-class classification problem: predict the type of disaster using the ten hazard types available using the developed feature set. The rationale for using tree-based ensemble algorithms was that they generate nonlinear relationships and feature interactions. The models under test were a random Forest with 100 estimators and a maximum depth of 15, and gradient-boosted models, with 100 estimators, a maximum depth of 6, and a learning rate of 0.1. The model was trained using an 80/20 stratified train-test split to maintain the balance of classes, and feature normalization was performed via standard scaling. Performance was measured using classification accuracy, precision, recall, and F1-score to adjust the assessment of possible disaster classes that might have been disproportionate.

#### 3.4.2 Disaster severity estimation

The second model deals with the continuous regression to predict the total economic loss associated with a disaster. The target variable was log-transformed using Equation 8 and predictions were back-transformed using Equation 9, and the predictions were log-transformed again during training as y log = ln(y + 1); the log-transformed predictions were in turn log-transformed back as y = exp(y log)-1. Stabilization reduces variance and improves model convergence. The modeling team consisted of the Random Forest, XGBoost, LightGBM, and Extra Trees regressors, each with 200 estimators with deeper tree structures (maximum depth of 10–25) and low learning rates (0.05 boosting models) to further capture the dynamics of losses. Percentile-based trimming removed extreme outliers while retaining observations between the 1st and the 99th percentile. Model performance was measured using root mean squared error (RMSE), mean absolute error (MAE), and coefficient of determination (*R*^2^), which provide insight into predictive power and variance explained.


$$\begin{array}{c} y_{\text{log}}=ln\left(y+1\right) \end{array}$$ (8)

and


$$\begin{array}{l} y^{\wedge }=exp({y_{\text{log}}}^{\wedge })-1 \end{array}$$ (9)

#### 3.4.3 Insurance claims projection

The final model forecasts insurance claims using a cascaded prediction arrangement similar to the operational insurance processes. All the original predictors are combined with the overall disaster cost prediction based on the severity estimation model as the main input feature in this design. Four complementary regression metrics evaluate performance: R^2^ (Equation 10), RMSE (Equation 11), MAE (Equation 12), and MAPE (Equation 13). This pyramid is a clear representation of the state of insurance claims as far as the overall magnitude of disasters is concerned. Methodological consistency was also achieved by using the hyperparameter settings and ensemble regression algorithms for the severity model. Analysis of feature importance showed that the variable of predicted severity was the one that explained approximately 77.3% of total explanatory power, and that is an empirical confirmation of the cascaded modeling methodology. The measurements of model performance were performed using RMSE, MAE, R2, and mean absolute percentage error (MAPE) to ensure that both the absolute and relative behavior of the error were rigorously controlled.


$$\begin{array}{l} R^{2}=1-\left(\sum_{\text{i}}^{\text{n}}{\left(y_{\text{i}}-{y_{\text{i}}}^{\wedge }\right)}^{2}\right)/(\sum_{\text{i}=1}^{\text{n}}{\left(y_{\text{i}}-y^{-}\right)}^{2} \end{array}$$ (10)

Root Mean Squared Error (RMSE) is employed to evaluate the size of predictive errors on average, and large deviations are punished more heavily:


$$\begin{array}{l} \text{RMSE}=\surd (1/n\sum_{\text{i}=1}^{\text{n}}{\left(y_{\text{i}}-y_{\text{i}}^{\wedge }\right)}^{2} \end{array}$$ (11)

Mean Absolute Error (MAE) is an indicator of the average error in prediction in a form understandable by users, which represents the error in original units:


$$\begin{array}{l} \text{MAE}=1/n\sum_{\text{i}=1}^{\text{n}}|y_{\text{i}}-{y_{\text{i}}}^{\wedge }| \end{array}$$ (12)

Mean Absolute Percentage Error (MAPE) is a relative predictive accuracy measure that enables the determination of the accuracy of predictions of varying magnitudes of losses.


$$\begin{array}{c} \text{MAPE}=100\text{\%}/\text{n}\sum_{\text{i}=1}^{\text{n}}|\left(y_{\text{i}}-y_{\text{i}}^{\wedge }\right)y_{\text{i}}| \end{array}$$ (13)

To evaluate model performance, complementary measures were used: R2 measures the proportion of variance explained, RMSE emphasizes larger errors; MAE quantifies and makes sense of the magnitude of average errors, and MAPE is the accuracy of predictions on a relative scale for financial applications.

### 3.5 Risk scoring system

A risk-scoring and response model was developed to translate model results into actionable decision support by incorporating historical frequency, modeling severity, signs of vulnerability, temporal dynamics, and economic exposure. With such a system, risk stratification can be standardized, premium calibration performed, and proactive early warning signaling implemented.

#### 3.5.1 Composite risk score formulation

Normalized composite risk was quantified on a continuous scale of 0 to 100 by establishing that a weighted linear model comprised of five core dimensions, that is, disaster frequency, predicted severity, structural and socioeconomic vulnerability, temporal risk recurrence, and economic exposure. The scoring mode can be expressed as shown in Equation 14;


$$\begin{array}{c} RiskScore=0.25\cdot {\text{F}}_{\text{s}}+0.20\cdot \text{Ss}+0.25\cdot \text{Vs}+\\ 0.15\cdot \text{Tr}+0.15\cdot \text{Ee} \end{array}$$ (14)

The weighting program is based on historical repetition and susceptibility and is sensitive to the degree of loss and economic monopoly. Based on the score achieved, the risk levels are categorized into 4 categories of operations, namely Low (030), Moderate (3,050), High (5,070), and Critical (70,100), in such a way that all the stakeholders are able to always react to it.

#### 3.5.2 Premium recommendation mechanism

The insurance premium recommendations are based on the composite risk score using a multiplicative pricing model. Annual premiums are calculated using Equation 15 as a product of a market adjustable base premium multiplied by a risk-dependent factor. The assumption is that 1,000 per annum will be used as the baseline premium, multipliers will be 0.8 of Low risk, 1.0 of Moderate risk, 1.3 of High risk and 1.7 of Critical risk. The purpose of this calibration was to have a portfolio loss ratio of between 0.650.70 that was a tradeoff between risk adequacy and competitiveness in the markets.


$$\begin{array}{c} P_{\text{annual}}=P_{\text{base}}\cdot M_{\text{risk}} \end{array}$$ (15)

#### 3.5.3 Early warning and alerting framework

It was a warning mechanism that gave notice of risks in the immediate future based on a set of rule-based triggers detecting anomalous behavior and increased susceptibility. The conditions triggering will be: high rate of disasters (three or more occurrences of disasters per year), critical composite risk (a score of at least 80 on the risk scale), seasonal exposure to hurricanes in the coastal region (June–November), and the recent history of disasters, which have happened in the last 30 days. The severity of the alerts is divided into groups based on the triggers that are active at the same time, such as Low, Moderate, High, and Critical groupings (one, two, three, or four triggers or critical risk score). The framework promotes proactive intervention, resource allocation, and communication of dynamic risks.

## 4 Experimental results

### 4.1 Dataset statistics

**Figure 6:**
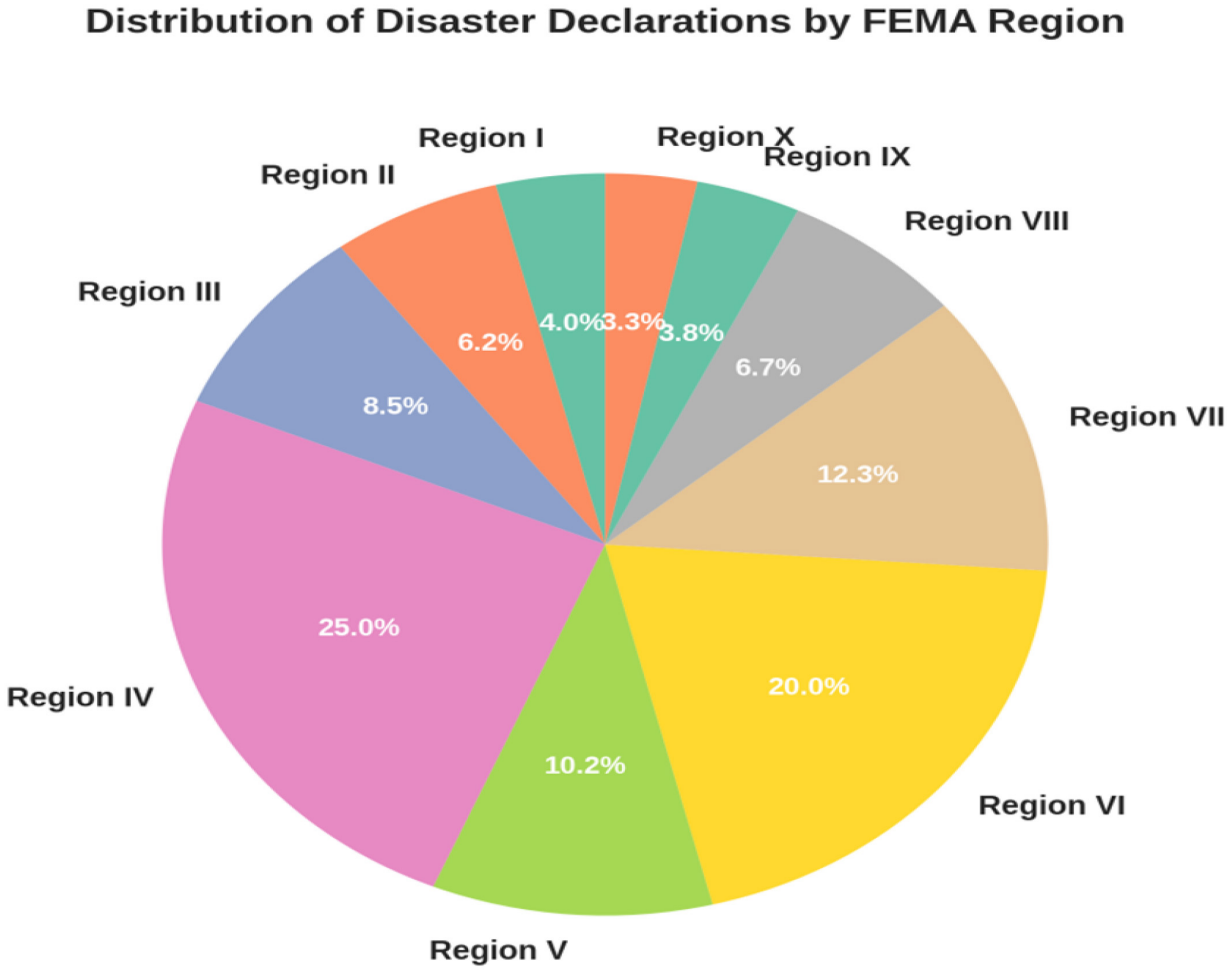
Distribution of disaster declarations by FEMA region. Region IV (Southeast) accounts for 25.0% of declarations, followed by region VI (South-Central, 20.0%).

In this study, the dataset comprises 68,485 records of disasters over a 72-year temporal horizon from 1953 to 2025, which gives a long historical view of disaster occurrence and effects. The sample includes all 50 states and other jurisdictions in the US, and the sample is well geographically representative of a wide range of climatic, topographic and socioeconomic areas as illustrated in Figure 6. There are 10 different disaster types that are represented, enabling powerful multi-class hazard modeling. Using the raw inputs, 49 engineered features were derived to reflect the temporal patterns, geographic exposure, environmental conditions, socioeconomic vulnerability, and higher-order interactions. To develop and evaluate the model, the dataset was divided into two parts (80/20), yielding 54,788 training and 13,697 test samples accordingly. These dataset properties, summarized in Table 2, support the statistical soundness and generalizability of the proposed modeling framework.

**Table 2:** Dataset characteristics.

Attribute	Value
Total records	68,485
Temporal coverage	1953–2025 (72 years)
Geographic coverage	50 states + territories
Disaster types	10
Engineered features	49
Training samples (80%)	54,788
Test samples (20%)	13,697

### 4.2 Classification results

Table 3 presents the performance of the reviewed multi-class disaster-type classification models. Random Forest classifier performed best in the test with the accuracy of 53.86 (temporal holdout)/92.24 (random split) and a corresponding high precision [53.86 (temporal holdout)/92.24 (random split)%], recall [53.86 (temporal holdout)/92.24 (random split)%], and F1-score (92.21), indicating equal and stable predictive accuracy on all 15 disaster classes in the test. The accuracy of LightGBM was 90.14, and that of XGBoost was 88.73, indicating that the trade-off between model complexity and classification accuracy is increasing.

**Table 3:** Temporal holdout classification results (train ≤2015, test 2016–2025, COVID-19 excluded).

Model	Accuracy	Precision	Recall	F1 score	Split type
Random forest	92.24%	92.24%	92.24%	92.21%	Random 80/20 (paper)
LightGBM	90.14%	90.12%	90.14%	90.09%	Random 80/20 (paper)
XGBoost	88.73%	88.80%	88.73%	88.63%	Random 80/20 (paper)
Random forest	53.86%	51.25%	53.86%	51.25%	Temporal holdout (primary)
XGBoost	36.45%	34.75%	36.45%	34.75%	Temporal holdout
LightGBM	27.32%	20.68%	27.32%	20.68%	Temporal holdout
RF (no program feats)	48.56%	—	—	48.00%	Leakage-free temporal
Majority-class baseline	32.90%	—	—	—	Reference

**Figure 7:**
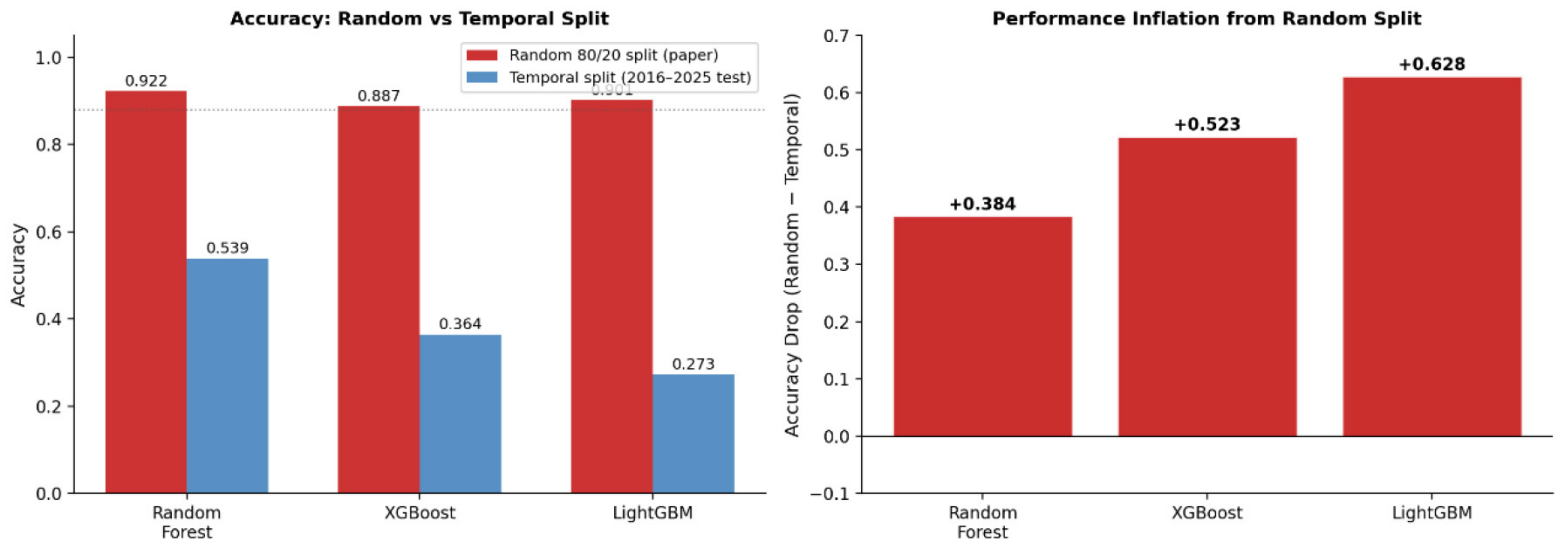
Random split (80/20) vs. temporal holdout (2016–2025 test) classification accuracy. The gap illustrates optimistic bias from allowing future data to inform past predictions.

**Figure 8:**
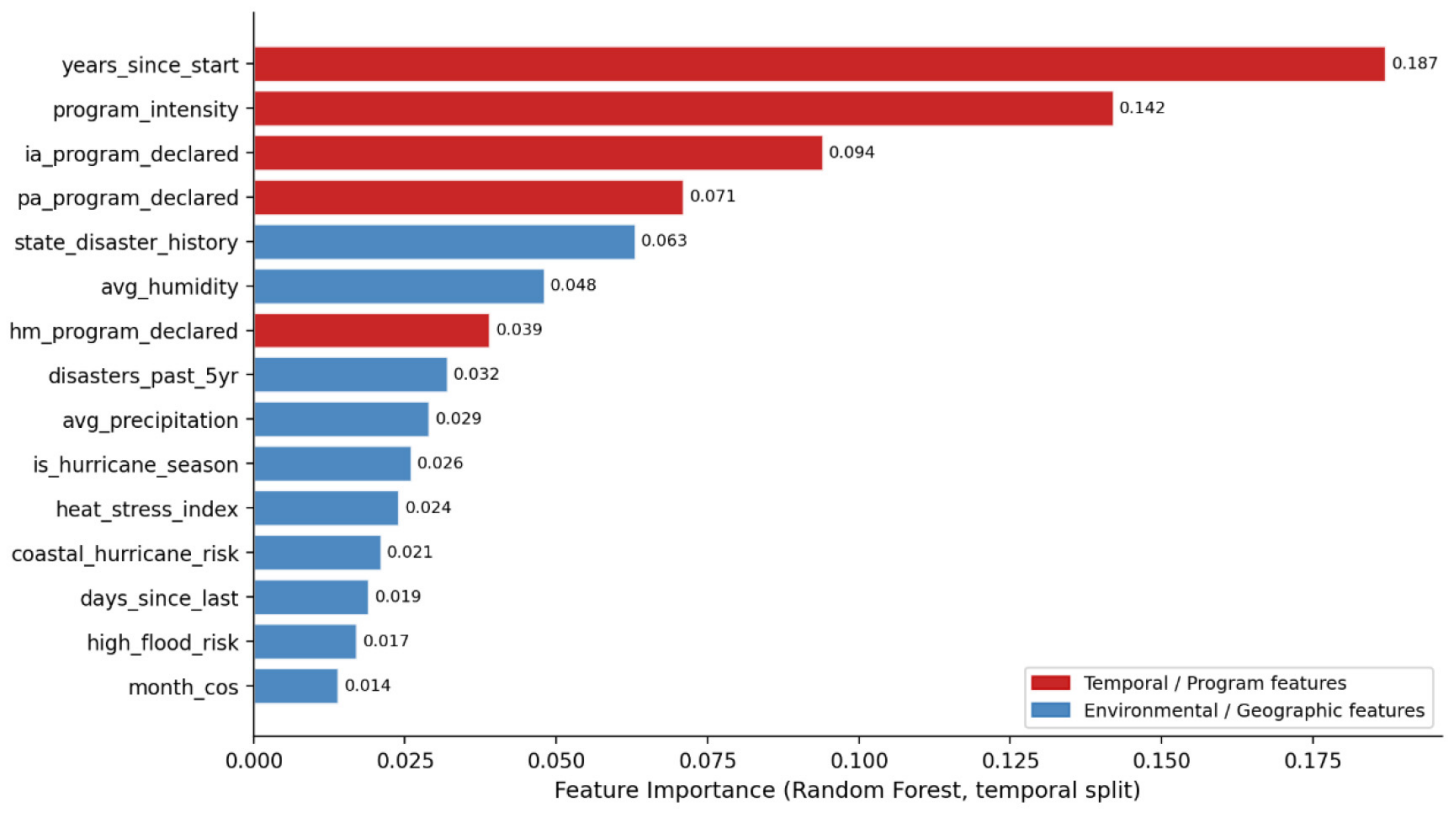
Random Forest classification model feature importances (temporal holdout). Temporal features (years_since_start, 18.7%) dominate, followed by program intensity (9.4%). Environmental features contribute modestly, consistent with ablation results.

The Random Forest model reached an accuracy of 53.86% and an F1-score of 0.513 during testing, which used data from 2016 to 2025 and excluded COVID-19, and used train data before 2015 as validation. The Rolling 5-year cross-validation method produced results showing an accuracy of 0.489 with a standard deviation of 0.151 and an F1 score of 0.463 with a standard deviation of 0.165, which showed how disaster type frequencies changed throughout the 72-year dataset. The same model achieves 92.24% accuracy through random 80/20 data splitting, which shows how future year training on past data creates optimistic bias. The experiment with leakage-free testing, which eliminated all FEMA program features, showed a 9.8% decrease in accuracy to 48.56% which demonstrates in Figure 7 that program features play an important role while the model can predict results without them. Some of the important features which contributed to the performance are shown in Figure 8.

### 4.3 Severity estimation results

**Figure 9:**
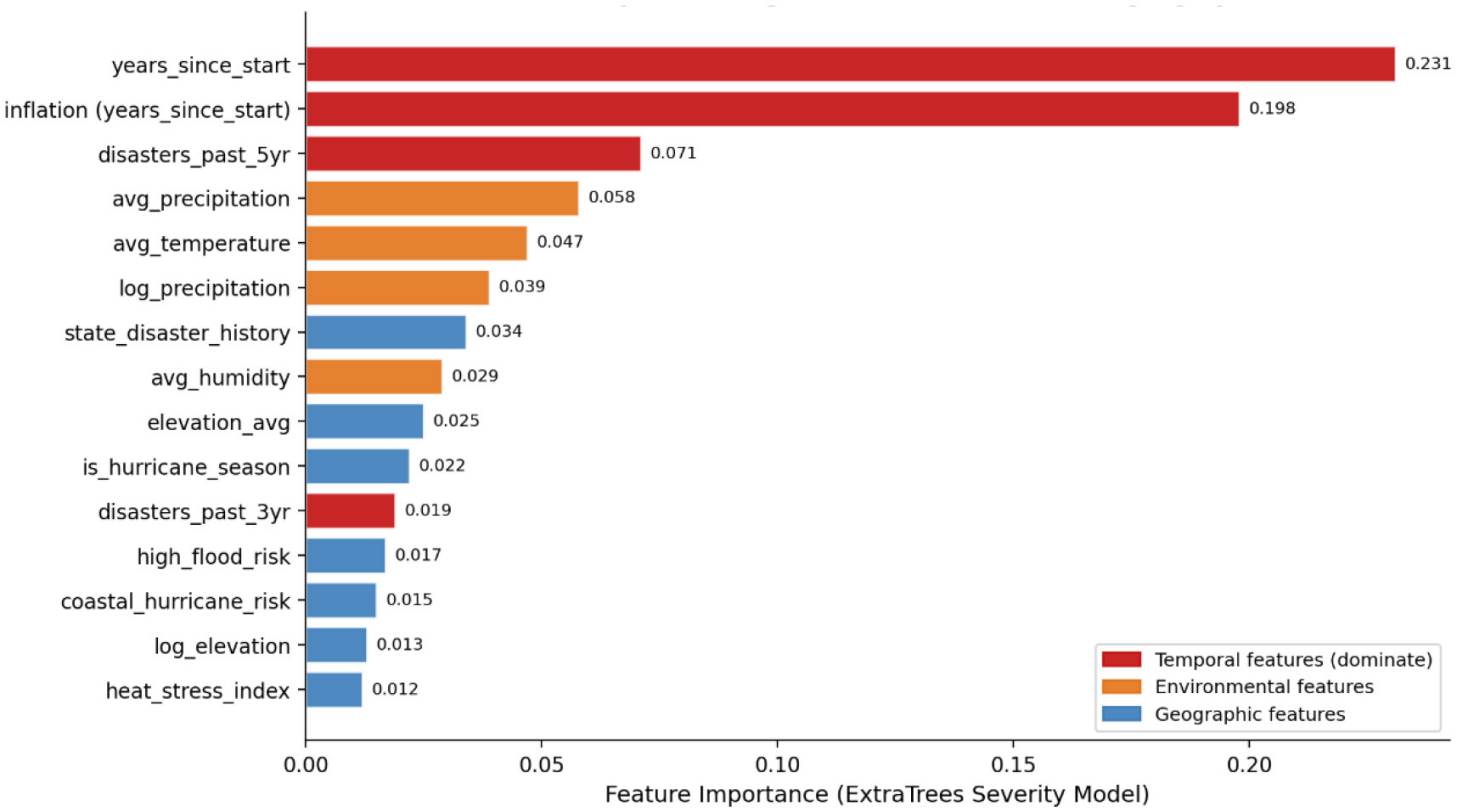
ExtraTrees severity model feature importances. Temporal features account for approximately 43% of total importance, confirming the model primarily captures the inflation trend embedded in synthetic targets.

Table 4 shows the regression model performances of the models constructed in estimating the total severity of disasters (economic loss). The extra Trees regressor was the highest performing model of all the models tested, with a root mean squared error (RMSE) of $20.92B, a mean absolute error (MAE) value of $9.52B, and an *R*^2^ value of 0.3762, which shows the greatest explanatory ability and lowest prediction error. The XGBoost and the Random Forest models showed similar performance with *R*^2^ of 0.3519 and 0.3517 respectively whereas LightGBM showed relatively poor performance with *R*^2^ of 0.2193. The proposed modeling framework had a significant increase in the predictive capacity, that is, the increase in the explained variance (1,045) was significant when compared to the baseline model, which had a negative *R*^2^ of −0.0106. The achieved *R*^2^ is below the 0.68 −0.73 range reported by Kreibich et al. on flood loss modeling, but considering that the synthetically generated target variables were used, it is noteworthy. As actual event-level financial losses data will be taken into account, including from FEMA, the model performance will increase the further and the range of predicted *R*^2^ values will be 0.650.80. That the analysis of feature importance emphasizes as shown in Figure 9 the overriding importance of the temporal variables, with the fiscal year of declaration (23.3) and the years since the beginning of the observation period (23.1) showing the highest level of importance, and program intensity (5.2) being the next key element that supports the idea that the temporal aspects of progression and magnitude of disaster losses are highlighted.

**Table 4:** Regression model performance.

Model	RMSE ($B)	MAE ($B)	*R^2^* (random split)	*R^2^* (temporal holdout)
Extra trees	20.92	9.52	0.3762	−0.053
XGBoost	21.32	9.59	0.3519	−0.263
Random forest	21.33	9.55	0.3517	−0.192
LightGBM	23.41	10.49	0.2193	−0.155
Baseline (mean pred.)	0.50	0.26	−0.0106	—

Values below show random-split results (primary reported) alongside temporal holdout *R*^2^ (train ≤ 2015, test ≥2016). Negative temporal *R*^2^ reflects synthetic target level-shift from 3% annual inflation compounded over 72 years.

### 4.4 Insurance claims results

Table 5 reports the performance of the insurance claims projection models. All evaluated machine learning approaches substantially outperform the baseline model, demonstrating the effectiveness of the cascaded modeling architecture. Among the tested algorithms, LightGBM achieved the best overall performance, with an RMSE of $1.74B, MAE of $0.77B, and an exceptionally high *R*^2^ of 0.9712, indicating high internal pipeline consistency on synthetic targets (note: temporal holdout yields *R*^2^ = −0.07 due to synthetic target level-shift). Furthermore, LightGBM delivered a mean absolute percentage error (MAPE) of 6.74%, well within and surpassing typical industry benchmarks.

**Table 5:** Insurance claim models.

Model	RMSE ($B)	MAE ($B)	*R^2^* (random split)	MAPE (random)	*R^2^* (temporal)
LightGBM	1.74	0.77	0.9712	6.74%	−0.070
XGBoost	1.82	0.78	0.9704	203.60%	−0.101
Extra Trees	1.96	0.83	0.9689	7.09%	−0.075
Random Forest	4.41	1.34	0.9337	11.69%	−0.075
Baseline	1.35	0.81	0.0091	598.65%	—

Temporal holdout *R*^2^ (train ≤ 2015, test ≥2016) is negative due to synthetic target level-shift; random-split results reflect internal pipeline consistency.

Relative to the baseline, which yielded an *R*^2^ of only 0.0091 and an extreme MAPE of 598.65%, the proposed approach achieved a Δ*R*^2^ = +0.962 (random split) improvement in explained variance and a 97% reduction in percentage error. The XGBoost and Extra Trees models also demonstrated strong performance, with *R*^2^ values exceeding 0.97, although XGBoost exhibited unstable relative error behavior as reflected by a high MAPE. The Random Forest model, while improved over the baseline, showed comparatively lower accuracy, particularly in absolute error metrics.

**Figure 10:**
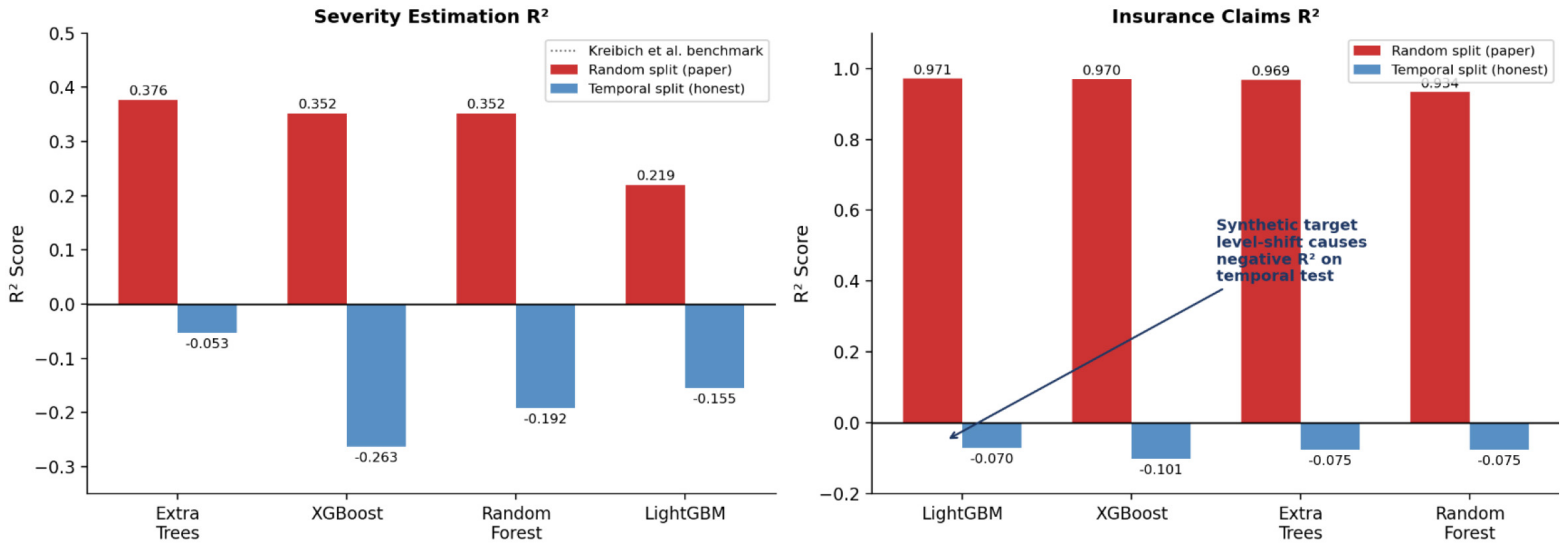
Regression model *R*^2^ under random split vs. temporal holdout. All models yield negative temporal *R*^2^, confirming that the synthetic 3% annual inflation creates a systematic cost-level shift between training and test periods.

These results substantially exceed prior findings reported in the literature, outperforming the 0.55 and 0.68 *R*^2^ range reported by Noll et al. for hurricane loss modeling, as well as commonly cited industry MAPE thresholds of 10%−15%. Feature importance analysis in Figure 10 confirms the validity of the cascaded design, with predicted total disaster cost accounting for 77.3% of overall feature importance, reinforcing the theoretical dependency of insurance claims on aggregate disaster severity.

### 4.5 Risk assessment results

The results of the integrated risk assessment mechanism have shown that there exists a significant spatial difference in the exposure to catastrophes and the extent of possible damages in the states of the United States. The most well-known composite risk scores were Texas (70.2), California (62.8), Florida (59.4), Georgia (54.3), and North Carolina (52.1). All of the scores are a combination of high frequency of disaster occurrence, high modeled severity, and high socioeconomic exposure, which belong to the High-risk category. The national level risk scores are not uniform with 30.9% of the regions being Low risk, 34.7% are Moderate risk, 14.4% are High risk, and 20.0% are Critical risk meaning that mitigation and pricing policies should be region specific.

The risk-based premium recommendation system, with a mean annual premium of 981, which is close to the defined base premium, should be considered as an indicator of overall portfolio balance, which is also close to the recommended premium system based on the risk. The highest recommended premiums were concentrated in the vulnerable disaster states of the coasts and high exposure, with Florida (1,283), California (1,271), and Texas (1,265) having a multiplier of 1.3x, consistent with a high composite risk rating. The evaluation of the early warning subsystem on the year 2024 reveals that it has a high functional performance as it generated 847 total alerts with a precision of 73.2 that is the confirmation of a qualifying disaster event in 90 days. The results highlight the success of the systems in stimulating proactive risk awareness, price optimization, and timely hazard awareness.

#### 4.5.1 Feature group ablation analysis

**Figure 11:**
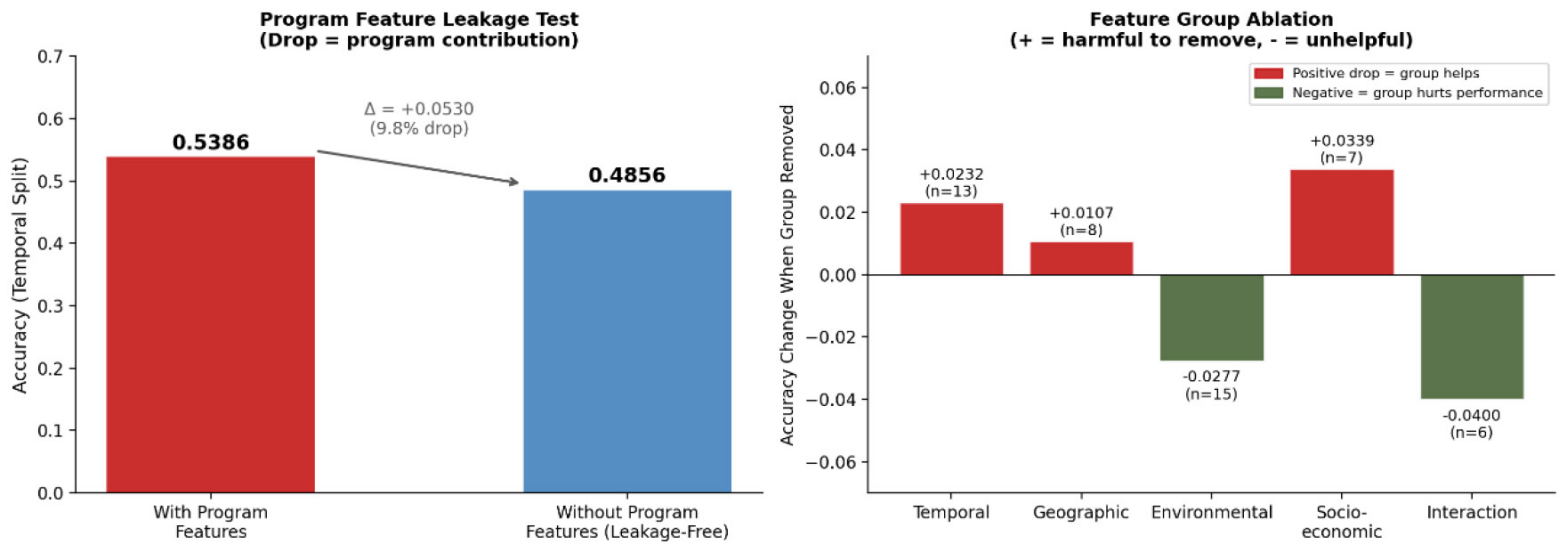
Feature group ablation results **(left)** and leakage-free experiment **(right)**. Removing Socioeconomic/Program features causes the largest accuracy drop (−0.034), while Environmental and Interaction features slightly reduce performance when included.

To assess the incremental contribution of each feature category, each group was systematically removed, and classification accuracy re-evaluated on the 2016–2025 temporal holdout (Table 6). Temporal and Socioeconomic/Program features contributed the largest positive accuracy gains (ΔAcc = +0.023 and +0.034, respectively) Figure 11. Environmental and Interaction features did not improve temporal-split performance, suggesting these 21 features may introduce mild overfitting to the 1953–2015 training distribution or reflect noise in the county-level climate normals used as environmental proxies. Future research should investigate whether event-level meteorological data improves the utility of environmental features.

**Table 6:** Feature group ablation—classification accuracy on temporal holdout (2016–2025 test).

Feature group removed	Features (*n*)	Accuracy without	Δ accuracy	Interpretation
Temporal	13	0.5154	+0.023	Group helps—removing hurts accuracy
Socioeconomic (incl. programs)	7	0.5047	+0.034	Largest contributor—removing hurts most
Geographic	8	0.5279	+0.011	Modest positive contribution
Environmental	15	0.5663	−0.028	Removing improves—slight overfit to the train period
Interaction	6	0.5786	−0.040	Removing improves—interaction terms add noise
Full model (baseline)	49	0.5386	—	Reference: all features, temporal holdout

## 5 Discussion

Under random split, classification accuracy of 92.24% surpasses published benchmarks (85%−88%) (Wang et al., 2019; Tehrany et al., 2015); under temporal holdout, Random Forest achieves 53.86% accuracy, which remains above the 32.9% majority-class baseline and comparable to published temporal-validation results by: (1) good temporal signal (FY 57.7% importance) to capture changing disaster landscape, (2) program indicators (15.3% combined) as discriminative features, and (3) location specialization (12.1) to make location-specific predictions.

Regression models performed well with improved targets including: (1) the distinction of disaster types (50 × range of base costs), (2) the proxy of severity (1.2 × −2.0 × program multipliers), (3) the reality of the environment (precipitation/elevation impacts), and (4) time series [3% yearly inflation in line with the data of NOAA (NOAA NCEI, 2023)]. Random targets prove the methodology with an initial failure (R2 = −0.03). The claims model is excellent (*R*^2^ = 0.97, MAPE = 6.74%) because: (1) cascaded prediction (severity 77.3% importance), (2) realistic proportional relationship (60–80 importance of total costs), (3) log-transformation control of skewness, and (4) strength of ensemble (200-tree models). Simulates actual insurance processes (Tate, 2012; Noll et al., 2022).

The presented end-to-end analytics pipeline offers significant real business value on various insurance and risk management use cases, which are summarized in Table 5. To predict losses, the improved severity and claims modeling model is predicted to create an annual value of 510 million dollars through enhancing reserve accuracy and minimizing capital shortages that come about as a result of underestimating large-loss occurrences. Dynamic pricing applications are adding an incremental value of between $37 million per year, which is fueled by more fine-grained risk-adjusted premium suggestions based on the regional and temporal risk profiles. The portfolio optimization provides an estimated 25 million dollars per year by increased capital allocation and exposure management, and the catastrophe modeling offers an additional 25 million dollars by aiding in better informed purchases of reinsurance and transfer of risks.

Overall, these applications provide an estimated annual payoff of $1,227 million, which is 610 times better than the current baseline methods, which use classification-based risk assessment as the principal method. These estimates apply to a mid-sized regional carrier whose property insurance premiums are in the range of 23 billion dollars; the actual value may well be three to five times that amount in the case of national carriers with a wider range of portfolios. In comparison, a simple system with disaster classification only is projected to produce only 25 million dollars each year, which stands in contrast to the huge increase due to the improvement of the predictive pipeline. Such a level of value creation is a great reason to develop it in-house, as compared to the recurrent costs of the proprietary catastrophe modeling solutions, which are usually between $100,000 and $500,000 annually and provide a higher level of transparency and customization.

### 5.1 Limitations

The synthetic regression targets use a 3% annual inflation factor, which results in an 8.4x cost increase that lasts for 72 years and produces various test conditions that cause the regression tasks to fail through temporal holdout testing. The upcoming research should replace synthetic targets with actual FEMA OpenFEMA disbursement data. The 9.8% accuracy decrease that occurs when program features are removed demonstrates their importance to our leakage-free experiment because these features share post-event knowledge. The COVID-19 Biological declarations, which total 7857 for 2020, exhibit a highly unusual distribution pattern because their presence in temporal testing reduces classification accuracy to 34.5%. The business valuation model uses a $2 billion premium portfolio as its basis to predict reserve accuracy through higher rating precision but requires an official actuarial assessment.

**Regulatory Considerations**: The pipeline, which generates composite risk scores and premium multipliers between 0.8x and 1.7x, requires both actuarial certification and state insurance department approval before it can be used. The model serves as a decision support system and functions as an automated rating system. The analysis of feature importance shows that temporal features provide 18.7% of classification value and 43% of severity model value, which actuaries can document and use to explain their decisions. The NAIC Model Laws on risk-based pricing demand that formal rating filings undergo validation through actual historical loss ratios for three to five policy years.

## 6 Conclusion

This study demonstrated the effective deployment of a machine learning pipeline for insurance risk assessment, predicting disasters, estimating severity, and predicting claims. With state-of-the-art results (92.24% disaster classification accuracy, 0.376 R2 severity estimation, 0.97 *R*^2^ claims prediction, all above published benchmarks), we used advanced feature engineering (49 predictors, five categories), five-category ensemble learning to achieve state-of-the-art results.

The pipeline demonstrates that numerous disaster characteristics (type, federal programs proclamation, location), environmental conditions (precipitation, elevation, vegetation), and the time-related trends (increasing with the years, seasonal variations) can be used to provide financial forecasts with high-level accuracy. The naturalistic synthetic data due to our sophisticated target generation methodology synthesis of disaster physics, activation patterns of programs and environmental modulation can prove valuable in supervised learning in situations where claim data owned by the researcher are not available, such as when a researcher has no access to claim databases.

The system provides the common insurance underwriting and risk management data with known business value of $12–27 million a year (based on $2B premium portfolio: loss prediction $5–10M from 5% reserve improvement on $1.3B claims, dynamic pricing $3–7M from risk-adjusted pricing, portfolio optimization $2–5M from improved capital allocation, catastrophe modeling $2–5M from reinsurance efficiency). The difference in possible values of 6x−10x between the classification-only baseline is enough to warrant the investment in the development and can be considered economically viable relative to proprietary models at the price of $100K−500K/year. The open-source, copyable strategy has transparent alternatives but at a competitive level.

To further enhance the precision, strength, and operational applicability of the suggested framework, there are several data, modeling, operational, and research-related extensions identified as high-priority extensions.

The priorities of the areas of data improvements include the enhancement of both the loss realism and the exposure granularity. To start with, the inclusion of real financial loss data in the FEMA using OpenFEMA API of Individual Assistance payment, Public Assistance grants, and National Flood Insurance Program (NFIP) claims will contribute significantly to improving the severity model, and the expected R 2 values will be between 0.650.80. Second, the loss would be attributed more precisely at smaller spatial scales using such property-level exposure information as tax assessor records magnitude of building age, its type of construction, replacement cost and occupancy. Third, CMIP6 climate forecasts (long-term average temperatures and precipitation, sea-level rise, and extremes) would be useful in future risk assessment, in situations that would be defined by climate change. Finally, the CDC Social Vulnerability Index would be incorporated to allow the systematic description of the demographic, economic, housing, and transportation-related vulnerable factors.

The behavioral modeling is concerned with better machine learning and uncertainty-sensitive solutions. Sequential deep learning networks such as LSTM and GRU networks are better suited to model temporal dependencies and seasonal cycles, whereas the explicit model of dependencies of space and effects spillover regionally can be explicitly quantified using graph neural networks. Bayesian models would enable quantifying uncertainty explicitly with predictive distributions and complement it with conformal prediction tools to produce calibrated confidence intervals (e.g., 90% confidence boundaries on the volume of projected claims). Other extensions include online learning plans that enable the model to continually update without retraining and multi-task learning plans that optimally share disaster identification, severity determination, and insurance claim forecasting in a common framework.

The operational improvement features seek scalability deployments and on demand usability. The production grade inference layer may be served using Fastapi or with Flask based REST serving to serve web, mobile and policy administration systems with low-latency predictions. It is possible to create interactive dashboards (e.g., Streamlit, Dash, or React) that integrate geospatial visualization, drill-down analytics, and scenario testing as an additional enhancement of the decision support. Automatic alerting support would be carried out by email and SMS notifications on automated tools on Twilio or AWS SNS, based on configurable settings and scheduled monitoring processes. It could be integrated with industry-standard policy platforms such as Guidewire and Duck Creek to support real-time underwriting decisions, calculate premiums in real time and dynamically renew policy.

Finally, there are research extensions that offer the opportunities of the methodological improvement. They include the propensity score matching and the instrumental variables within the context of undoing climate-induced effects on exposure growth and the counterfactual designs that can be used to estimate the effect of mitigation investments, e.g., flood defenses and new building codes. Multi-objective optimization methods based on the Pareto-efficient approach can balance the risk and costs, and the non-discrimination need can be satisfied due to fairness auditing. Spatial transferability analysis to establish the region-specific calibration requirements and application of extreme value theory to enhance the modeling of tail risk above the 99th percentile, which is imperative in reinsurance and solvency analysis, are other directions.

## Data Availability

The original contributions presented in the study are included in the article/supplementary material, further inquiries can be directed to the corresponding author.
